# Supplementary material for: Symmetry breaking in drop bouncing on curved surfaces
Source: Nat Commun. 2015 Nov 25;6:10034. doi: 10.1038/ncomms10034 (PMC4674688; doi:10.1038/ncomms10034)
Supplement: Supplementary Information — Supplementary Figures 1-5, Supplementary Table 1 [file ncomms10034-s1.pdf]

Supplementary Figures

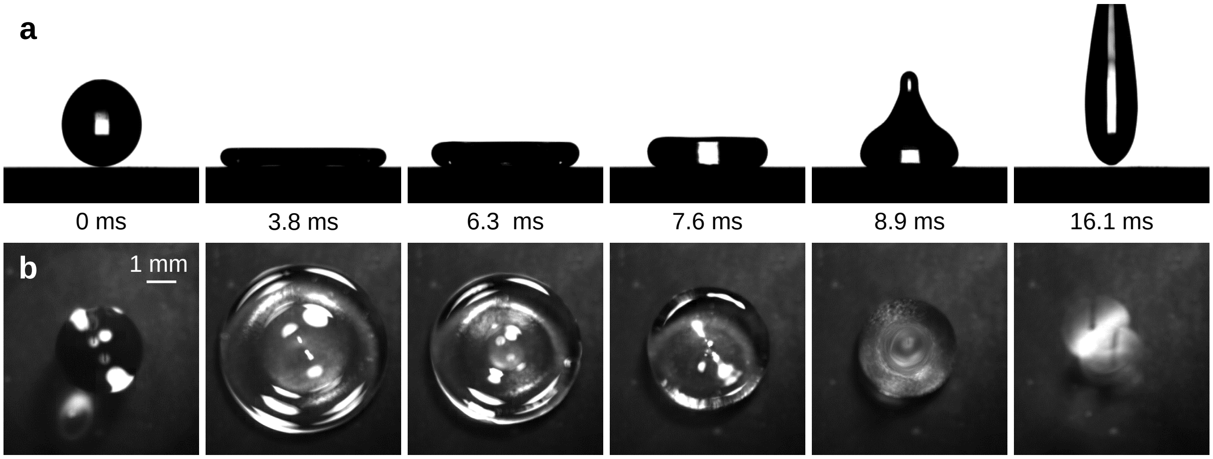

Supplementary Figure 1 |. **a**, Selected high-speed images captured from the side show the bouncing dynamics of a water drop hitting a flat (symmetric) surface with the same coating as the asymmetric surface. The drop detaches from the surface at 16.1 ms ($= 2.48\sqrt{\rho r_0^3/\gamma}$ with drop radius $r_0 = D_0/2 = 1.45\,\mathrm{mm}$ and impact velocity $v_0 = 0.63\,\mathrm{m\,s^{-1}}$). **b**, Simultaneous plan-view images demonstrate that the drop bouncing dynamics is symmetric.

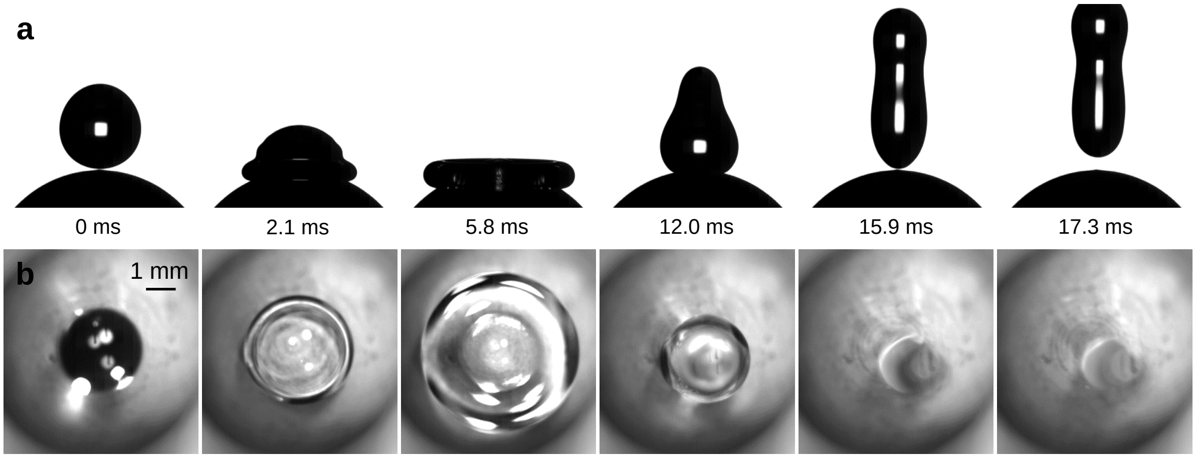

Supplementary Figure 2 |. Selected snapshots of a drop hitting the top of a spherical (symmetric) superhydrophobic surface. The diameter of the sphere is 8 mm. **a**, High-speed images captured from the side reveal that the contact time is 15.9 ms ($= 2.45\sqrt{\rho r_0^3/\gamma}$ with drop radius $r_0 = D_0/2 = 1.45\,\mathrm{mm}$ and impact velocity $v_0 = 0.63\,\mathrm{m\,s^{-1}}$). This time is close to that on the flat surface, as expected for any impact where the flows have circular symmetry. **b**, Simultaneous plan-view images show that the drop retains a circular symmetry during the entire impact process.

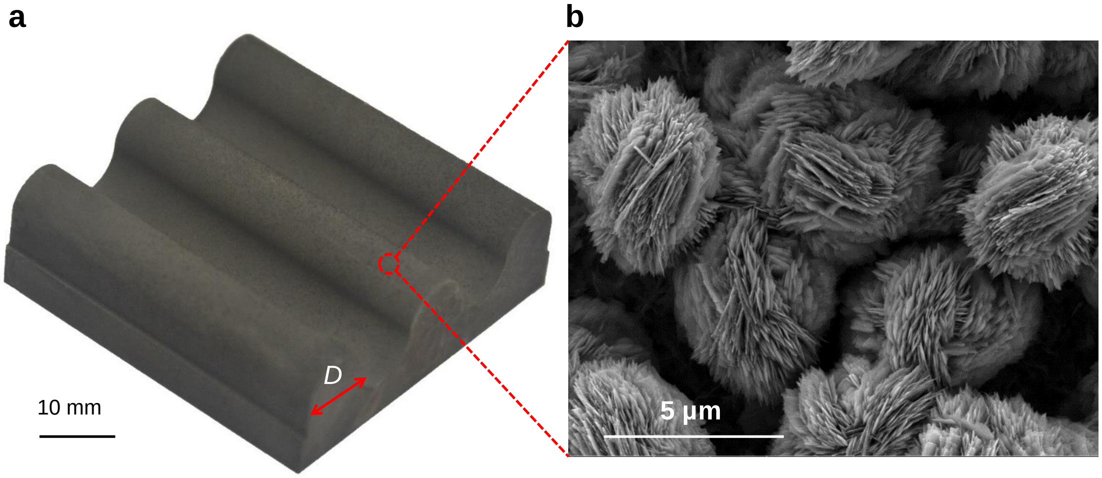

Supplementary Figure 3 |. **a**, Geometry of the fabricated asymmetric surface (optical image) with diameter of curvature $D = 8$ mm. **b**, SEM image showing the surface was uniformly coated with hydrophobic flowers of dimension $\sim 20\,\mu$m to render it extremely water-repellent.

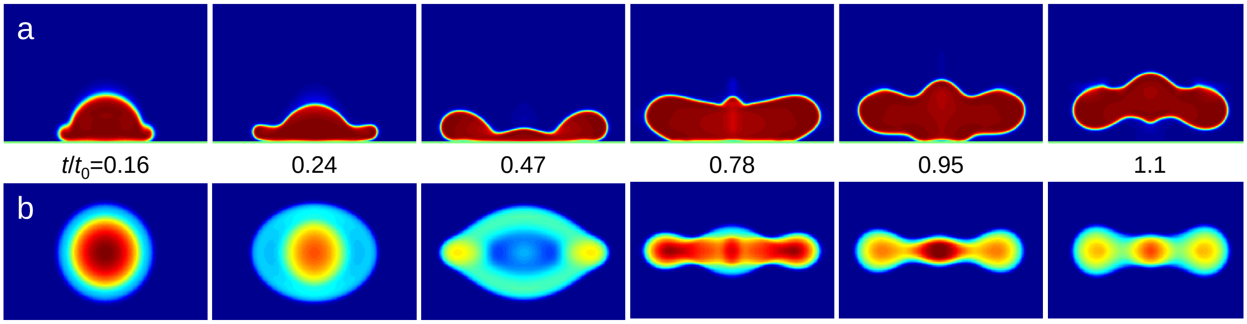

Supplementary Figure 4 |. Side view (**a**) and plan view (**b**) of the time evolution of a drop bouncing on a flat surface with manually imposed momentum asymmetry. Shortly after impact the momentum along one axis was doubled whilst that that along the perpendicular axis was halved. The subsequent behaviour is qualitatively similar to that seen on the asymmetric surface. The parameters used in this simulation are given in Supplementary Table 1.

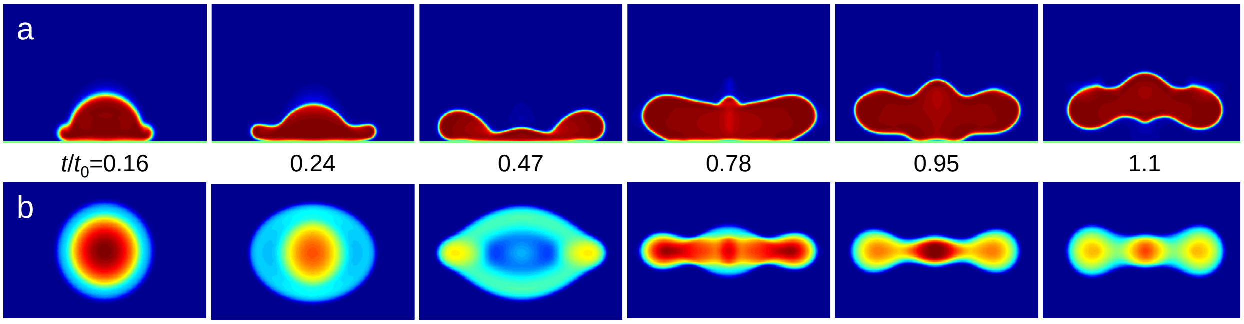

Supplementary Figure 5 |. Selected snapshots of the (**a**) side view and (**b**) plan view of an elliptical drop bouncing on a flat surface. The subsequent behaviour is qualitatively similar to that seen on the asymmetric surface. The parameters used in this simulation are given in Supplementary Table 1.

Supplementary Table

Quantity	Value (lattice units)
Surface tension	0.0075
Liquid dynamic viscosity	0.0042
Gas dynamic viscosity	0.000082
Liquid density	1
Gas density	0.0012
Interface width	9
Contact angle	160
Initial velocity	0.04
Drop radius	50
Mobility	0.94
Gravitational constant	0
Weber number, *We*	10.7
Ohnesorge number, *Oh*	0.0068
Bond number, *Bo*	0

Supplementary Table 1. Parameters used in the simulations, and the corresponding dimensionless variables.
